# Adaptation of a Fungal Pathogen to Host Quantitative Resistance

**DOI:** 10.3389/fpls.2018.01554

**Published:** 2018-10-31

**Authors:** Lise Frézal, Guy Jacqua, Claire Neema

**Affiliations:** ^1^INRA-URPV, Guadeloupe, France; ^2^CNRS – ENS – INSERM, Institut de Biologie de l’Ecole Normale Supérieure, Paris, France; ^3^UMR BGPI, Montpellier SupAgro, Campus International de Baillarguet, Montpellier, France

**Keywords:** local adaptation, aggressiveness, quantitative resistance, *Colletotrichum gloeosporioides*, *Dioscorea alata*

## Abstract

Impact of host quantitative resistance on pathogen evolution is still poorly documented. In our study, we characterized the adaptation of the pathogenic fungus *Colletotrichum gloeosporioides*, to the quantitative resistance of its host, the water yam (*Dioscorea alata*). Genetic and pathogenic diversities of *C. gloeosporioides* populations were specified at the field scale. We used nuclear markers to describe fungal population structuring within and between six fields of three cultivars differently susceptible to the fungus. Strain aggressiveness was then quantified in the laboratory through cross-inoculation tests. The high level of genetic diversity and significant linkage disequilibrium revealed a significant influence of clonal reproduction in the *C. gloeosporioides* evolution. The recorded fungal migration between fields was weak (evidence for a dispersion mode via tubers rather than splashing dispersal), which provides the first molecular evidence for limited *C. gloeosporioides* migration via yam tuber exchanges. *C. gloeosporioides’s* populations are adapted to their host resistance. The aggressiveness of the fungal clones seems to have evolved toward an accumulation of components specific to each host cultivar. Despite the remaining marks of adaptation to the former widely cultivated host, adaptation to current cultivars was clearly depicted.

## Introduction

Discernment on the adaptive potential of plant pathogen species is essential to designing sustainable integrated pest management framework in which, durability of crop resistance is one crucial point. The adaptive potential of plant pathogen species highly depends on the forces shaping the pathogen evolution. Among these forces, the migration (spatial dispersal), the recombination (reproductive mode), genetic drift and above all, the selection by the host-plant resistances, are of particular importance (McDonald and Linde, 2002). Pathogen populations can either adapt to the most common host (general adaptation), or be divided into several entities, each adapted to one particular host genotype (local adaptation, Gandon et al., 1996; Gandon and Van Zandt, 1998). The theory of polygenic traits’ evolution under divergent selection has not been much investigated, especially while investigating host pathogen interactions (Kawecki and Ebert, 2004). The few studies focusing on the host quantitative resistance impact on pathogen populations reported either the local adaptation of pathogen populations (Kaltz and Shykoff, 1998; Zhan et al., 2003), or a directional selection toward an increase in pathogen aggressiveness on all host cultivars (Andrivon, 1993; Andrivon et al., 2007; Montarry et al., 2007). In the present study, we described the population diversity of the plant pathogen *Colletotrichum gloeosporioides* in the Guadeloupean (French West Indies) agrosystem. We also investigated the pathogen aggressiveness as an adaptive trait of the fungi to the quantitative resistance of its host, *Dioscorea alata*.

The filamentous ascomycete and plant pathogen *C. gloeosporioides* (Penz.) Penz. & Sacc. [telemorph: *Glomerella cingulata* (Stonem.) Spauld. & Schrenk] causes anthracnose disease on various crops (Bailey and Jeger, 1992; strawberry, Freeman et al., 1998; olive, Talhinhas et al., 2005; avocado, mango, stylo, Chakraborty et al., 1999; ornamental lupinines, Elmer et al., 2001; *Salsola tragus*, Berner et al., 2006; peach tree, Kim and Hong, 2008), among which the water yam (*D. alata*). The life cycle of this haploid and hemibiotroph fungus includes both sexual and asexual reproduction. If the two reproductive modes are observed on water yam, the asexual mode prevails during yam anthracnose epidemics. Conidia (asexual spores) disseminate from plant to plant by wind-directed-rain splashing (Green and Simons, 1994; Penet et al., 2014) which limits dispersal to several meters from the source (Ntahimpera et al., 1999).

Sexual reproduction can nevertheless occur at the end of epidemics, on dead leaves or stems, which—when left on the soil surface—may contribute to the primary infection of yam (Ripoche et al., 2007). Ascospores (sexual spores) are produced in dried-perithecia that can eventually be wind-dispersed on a long distance (Abang, 2003). Besides, *C. gloeosporioides* generates quiescent infections in yam tuber epidermis (Simons and Green, 1994); As farmers traditionally save tuber portions from a previous harvest for the new crop propagation, mycelia can pass from one crop season to the next. Thus, commercial exchanges of yam tubers and quiescent infections confer to *C. gloeosporioides* a capacity for long-distance migration and for clonal propagation (Green, 1994).

*Dioscorea alata* foliar anthracnose has been reported in Oceania and India (Winch et al., 1984; McDonald et al., 1998), in the West and Central Africa (Asiedu et al., 1998; Mignouna et al., 2001) and in the Caribbeans (Coursey, 1967; Green and Simons, 1994; Cowger and Mundt, 2002). In the West Indies, anthracnose disease became the most damaging disease on water yam at the end of the 1970s (McDonald et al., 1998) and was associated with serious yield losses reaching 80–100% for the common cultivars (*cv*) of this time, i.e., *cv* Pacala, in Guadeloupe (Green and Simons, 1994). In the 1970s, the cultivar Plimbite was introduced in the Guadeloupean islands and has been extensively cultivated until it became as susceptible to anthracnose as *cv* Pacala. After the late 1990s, farmers started to use more resistant cultivars such as *cv* Kabusah and *cv* Tahiti, but kept planting previous cultivars (Penet et al., 2016).

Genetic determinants of the interaction between *C. gloeosporioides* and *D. alata* are poorly understood. For a long time, this resistance was thought to be polygenic (Green and Simons, 1994; Abang et al., 2001). More recently, Petro et al. (2011) work based on the Boutou and Pyramide *D. alata* cultivars provided strong evidence for the quantitative inheritance of the components associated with the *D. alata* resistance to *C. gloeosporioides*. Next to this quantitative resistance, a gene-for-gene-like component (Flor, 1971) of the interaction between *C. gloeosporioides* and *D. alata* cultivars has been unraveled (Mignouna et al., 2002; Petro et al., 2011). For instance, the single dominant locus (*Cdg1*) explains the specific resistance of *cv* TDa-95/00328 to moderately virulent *C. gloeosporioides* strains.

The genetic structure of *C. gloeosporioides* populations, causing yam anthracnose, has been mostly studied in Nigeria (Abang et al., 2001, 2004, 2005, 2006), which is the first yam producer in the world (71.5% of the world production, FAOstat 2007). There, *C. gloeosporioides* was shown to exhibit high genetic and phenotypic diversity from the lesion to the region scales (morphotypes, Vegetative Compatibility Groups, RAPD, MP-PCR) and a weak population structure according to the host or to the agroecological areas (Abang et al., 2004, 2006).

Yam breeding and the management of yam resistances in the French West Indies require knowledge about the influence of the migration, the reproductive mode and the yam quantitative resistance on *C. gloeosporioides* evolution. Moreover, the impact of the host quantitative resistance on the evolution of the pathogen aggressiveness has never been investigated. Aiming to document a part or all of these aspects, the objectives of this work were: (i) to estimate genetic diversity and its distribution among fields with neutral markers (AFLP); (ii) to infer the influence of recombination at the field scale; (iii) to study how the degree of host quantitative resistance to anthracnose influences the distribution of fungal aggressiveness diversity.

## Materials and Methods

### Fungal Sampling and Isolation

**FIGURE 1:**
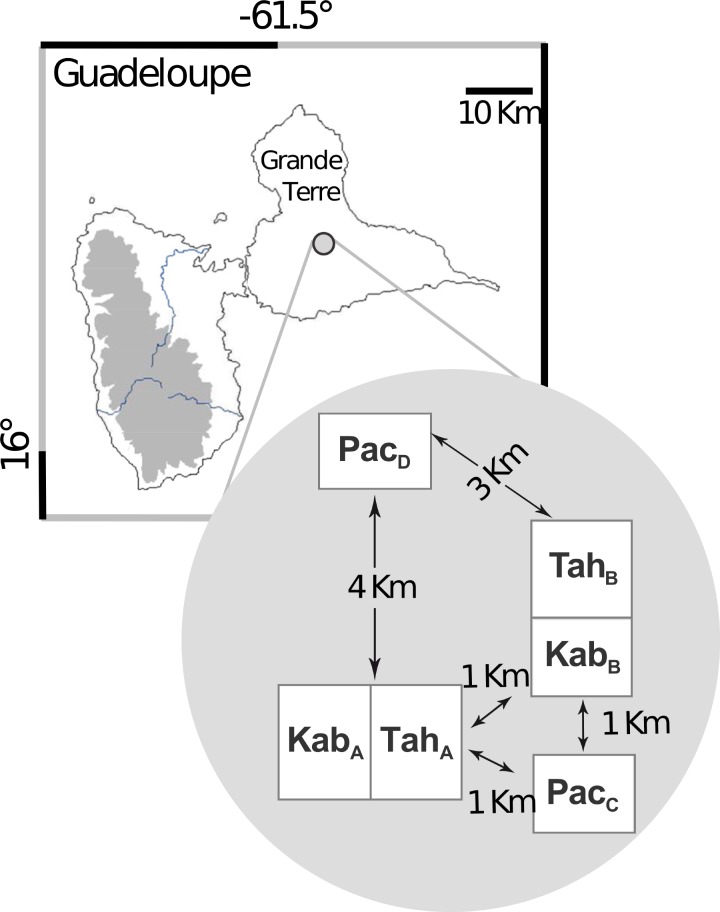
The locations of *Colletotrichum gloeosporioides* sampling sites in Guadeloupe (French West Indies); The relative geographical positions of the six yam (*Dioscorea alata*) fields are represented in the gray circle. The hosts cultivars were *D. alata cv* Kabusah (Kab), *cv* Pacala (Pac) and *cv* Tahiti (Tah). A, B were two locations where two fields were contiguous.

A total of 222 *C. gloeosporioides* isolates were collected from December 2001 to January 2002, from six yam fields within a 5-km-diameter area of the Guadeloupe (FWI, between Morne à l’eau and Le Moule, 16°15N, 61°35O, Figure 1 and (Supplementary Table S1). Sampling was done on three yam (*D. alata*) cultivars with different levels of resistance to anthracnose: *cv* Pacala (highly susceptible), *cv* Kabusah (moderately susceptible) and *cv* Tahiti (resistant). The study was carried out in six production fields of approximately 0.5 ha, with identical soil composition, topology, crop management and climate (Supplementary Table S1). One month before harvest, leaves with typical anthracnose lesions were collected from 50 plots per field, each distant from 2 to 5 m. Fungus isolation was performed from freshly harvested infected yam leaves (on the same day). Circa 1 mm^2^ of typical anthracnose lesions were plated on potato dextrose agar medium (PDA). *C. gloeosporioides* isolates were identified according to morphological characteristics (Sutton, 1992), then monoconidial cultures were obtained. The strains were grown on PDA-medium under photoperiodical conditions of 12 h day at 25°C and 12 h night at 23°C. Spores of 7-day-old cultures were suspended in distilled water to reach concentrations of 5.10^5^ conidia per mL. Mycelia were dried, thrown in liquid nitrogen and stored at −80°C for DNA extraction.

### DNA Extraction and AFLP Markers Genotyping

One milliliter of a suspension at 5.10^5^ conidia per mL was mixed with 30 mL of potato dextrose broth medium (PDB). After 72 h of incubation at 27°C, the mycelium was harvested, dried on sterile filter paper and stored at −80°C. Total genomic DNA was extracted from frozen mycelium with a phenol/chloroform protocol as described in Villaréal et al. (2002). DNA quality was checked by electrophoresis on 0.8% agarose gel and stored at −20°C in TE 0.1 (10 mM trisHCL, pH8; 0.1 mM EDTA). DNA digestion (*Mse*I and E*co*RI), ligation, pre-amplification, selective amplification and electrophoresis were conducted as described in Villaréal et al. (2002). The primer combinations used for selective amplifications were M*se*I-0 +AT/E*co*RI-0 +AT, M*se*I-0+ AG/E*co*RI +AG and M*se*I-0 +TA/E*co*RI-0 +CAA. The 6%-bisacrylamide-urea gel was stained with silver nitrate as described in Chalhoub et al. (1997). Polymorphic bands between 100 and 500 pb were manually referenced as present (“1”) or absent (“0”), which gave a 0–1 combination for each isolate. The AFLP patterns were checked for reproducibility with the two DNA replicates extracted and only clearly repeatable bands were used for further analyses. Co-migrating bands were assumed to be the same locus, and isolates having the same AFLP-pattern were assumed to be one single haplotype.

### Plant Material, Inoculation and *in vitro* Aggressiveness Assessment

In order to study the adaptive patterns of *C. gloeosporioides* on its host cv of origin, cross-infection experiments were performed. The diversity for aggressiveness was investigated on a 45-strain subsample composed of six to nine strains per field, randomly chosen in a cloned-corrected sample (i.e., sample containing only single-copy AFLP patterns).

### Host-Plant Material

*Colletotrichum gloeosporioides* strain aggressiveness was tested against the three host-plant cultivars from which they were isolated, i.e., water yam *cv* Pacala (high susceptibility), *cv* Kabusah (moderate susceptibility), *cv* Tahiti (low susceptibility), and also tested on one cultivar of reference for susceptibility to anthracnose, *cv* Plimbite (high susceptibility). Plants were grown from tuber pieces. All the yam tubers were produced in the INRA-URPV experimental center (Guadeloupe, French West Indies) which ensured the origin of the tubers as well as the sanitary control of their production. A total of 15-g tuber pieces were dipped for 24 h into fungicides (Chlorothalonil 550 g L^−1^ and Carbendazim 100 g L^−1^ supplied by Bayer CropScience), air-dried and covered with wood ashes. Tuber pieces were planted into humidified sterile compost in individual pots and put in a greenhouse under the natural conditions of Guadeloupe at the beginning of the wet season (25–30°C, >75% humidity, 12 h-photoperiod). To prevent any leaf infection on the plants used in the *in vitro* tests, we applied pesticides (oxythioquinox at 10 mg/mL supplied by Bayer CropScience) to the compost every 3 weeks until the first leaf emergence. As advised by Simons and Green (1994), fully expanded young leaves were harvested on 3-month old plants. Leaves were then washed with sterilized water and their adaxial surfaces were placed on moistened filter-paper disks in Petri dishes (one leaf per petri dish). Leaves were inoculated on the entire abaxial surface, as described by Toribio and Jacqua (1978).

*Inoculation* Strains were grown on PDA-medium in 12 h day at 25°C and 12 h night at 23°C conditions. Spores of 7-day old cultures were suspended in distilled water to raise concentrations of 5.10^5^ conidia mL^−1^. For each strain, 22.5 mL of the spore-suspension were sprayed on freshly cut leaves of each cultivar (five leaves per cultivar) using ecospray^®^ atomizer (LCF labo chimie France, Aix en Provence). At the same time, non-inoculated leaves (sprayed with water) of each cultivar were used as controls. Leaves were incubated at 100% humidity and in photoperiodical conditions of 12 h day at 27°C, 12 h night at 22°C. To reproduce the natural conditions of infection of *D. alata* with *C. gloeosporioides*, inoculations were carried out just before night. For all host-strain combination and condition (control and inoculation), five independent repeats were performed simultaneously.

*Disease assessment* two different symptoms are commonly observed when inoculating *D. alata* leaves with *C. gloeosporioides*: the typical lesion (large necroses, dark brown, coalescent, high expansion rate) and the pinpoint lesion [punctual necroses of less than 2 mm in diameter, black, not coalescent; Winch et al. (1984)]. Percentages of healthy, typical lesion and pinpoint lesion areas were visually estimated from the first to the 11th day after inoculation. In order to estimate strain aggressiveness, we use the analytical framework detailed in Frézal et al. (2012), which transforms the evolution of disease severity over time into one index, Ag. The construction of the Ag index is based the analysis of the symptom dynamics using a linear mixed-effects regression (PROC MIXED procedure; Laird and Ware, 1982) with two-knots, using SAS software (SAS Institute, 1987). The model is defined with a starting point at day 0 and two changes in slope (at day 6 and day 9). The typical lesion (Na) and total diseased areas (Da) were then defined as:

$$\mathrm{Da}(t)=\alpha_{\mathrm{Da}}\cdot t+\beta_{\mathrm{Da}}(t-6)+\gamma_{\mathrm{Da}}(t-9)$$

$$\mathrm{Na}(t)=\alpha_{\mathrm{Na}}\cdot t+\beta_{\mathrm{Na}}(t-6)+\gamma_{\mathrm{Na}}(t-9)$$

where, (*t*−6) = (*t*−6) if (*t*−6) > 0, and 0 otherwise; where (*t*−9) = (*t*−9) if (*t*−9) > 0, and 0 otherwise.

Strain aggressiveness index on each cultivar was defined as:

Ag=310⋅100((αDa6100)14⋅Da(day 11)100)+710⋅100((αNa⋅6100)14⋅Na(day 11)100)

For each strain, four indices were calculated: Ag-K on yam *cv* Kabusah, Ag-P on *cv* Pacala, Ag-T on *cv* Tahiti and Ag-Pl on *cv* Plimbite. We also focused on the initial lesion expansion rates (i.e., α_Na_) and the typical lesion area on day 11 (Na) as this provides detailed insight on strain fitness and helps to imagine hypothetic outcomes of the competition between strains.

### Data Analysis

#### Population Genetics Analyses

Diversity at each locus was estimated within subset of isolates grouped by field or cultivar. Unbiased gene diversity (Hd; Nei, 1987) was computed using GENETIX 4.05 (Belkhir et al., 1996/2004) on data sets containing only one copy of each haplotype per subset (i.e., clone-corrected data sets) (Chen and McDonald, 1996). Frequencies at each locus were estimated from each subset and used to calculate standard population statistics. In order to see whether the number of AFLP markers used in this study was sufficient to recover the maximal genotypic diversity, we plotted the genotypic diversity calculated from the multilocus genotype frequencies (Nei, 1987) versus the number of loci: our 23 polymorphic loci were discriminating and powerful enough (Supplementary Figure S1).

A minimum spanning tree based on the 23 ALFP markers was constructed for the full sample (222 isolates) using Bionumerics 5.1 with 100,000 resampling and partitioning of two majority trees. To determine population subdivision without any *a priori* knowledge, a second analysis was performed on the clone-corrected dataset using the Bayesian clustering method implemented in the software Structure version 2.3.3 (Pritchard et al., 2000). We used admixture and non-admixture models to identify genetic groups, both with distinct allele frequencies (Falush et al., 2003), and we assumed uniform priors for the vector of proportion (qi) of the individual i’s genome in each cluster. The scores of individuals in the genetic groups (i.e., the posterior estimates of the qi) correspond to the probability of ancestry in each one of them. We varied *K* from 1 to 10, with 10 replicates for each *K*, and with each simulation consisting in 800,000 Monte-Carlo Markov Chain (MCMC) iterations preceded by a burn-in period of 200,000 iterations. The most probable structure was determined by computing the posterior probability for each *K* using the distribution of maximum likelihoods and Δ*K* distribution (Evanno et al., 2005; Supplementary Figure S2), which is a quantity, related to the second order rate of change of the log probability of data with respect to the number of genetic clusters. Finally, to determine population subdivision without any *a priori* knowledge and without making any assumptions regarding the population genetics model, we described the genetic clusters from the clone-corrected dataset using the discriminant analysis of principal components (DAPC, Jombart et al., 2010) implemented in the R package adegenet 2.1.1 (Jombart and Ahmed, 2011; R Development Core Team, 2015).

Pairwise Weir and Cockerham (1984) values were calculated between all pairs of populations using GENETIX 4.05 (Belkhir et al., 1996/2004). Populations were defined as field samples and as subsets of individuals collected on the same yam cultivar, i.e., Kabusah, Tahiti and Pacala. Then, the significance of the pairwise-Fst was tested by 10,000 random permutations of the haplotypes (i.e., individuals) between the populations using Multilocus 1.3b software (Agapow and Burt, 2001).

The number of genotypes (Gs) was calculated in the whole sample and per filed from the clone-corrected dataset. As recommended by Arnaud-Haond et al. (2007) we calculated the modified index of clonal diversity, *R* from Dorken and Eckert (2001) as

_R=(G−1)/(N−1)_, with *G*, the number of identical multilocus genotypes and *N*, the number of individuals.

The degree of association between loci was estimated on the clone-corrected dataset by using the index of association, which is the ratio between the observed variance of the number of differences between pairs of strains and the expected variance under the hypothesis of absence of linkage disequilibrium (LD) (Agapow and Burt, 2001). We used a modified version of this index (*r*_D_), corrected for the dependence to the number of loci used. *r*_D_ has an expected value of 0 if there is no association of alleles at unlinked loci, as is expected in a randomly mating population. The significance of *r*_D_ was tested by a randomization procedure (1,000 times) by comparing the observed value to that expected under the null hypothesis of complete random mating (Agapow and Burt, 2001). Value calculation and tests were performed by Multilocus 1.3b software (Agapow and Burt, 2001). To prevent any effect of the population structure or of the LD between markers, we tested the estimated and tested (LD) between markers and retained 16 loci with no residual LD. We estimated and tested the significance of *r*_D_ within each genetic cluster using the 16 loci clone-corrected dataset.

#### Aggressiveness Analysis

Analyses of variance were conducted for the factors host cultivar, field of origin, genetic group using the General Linear Models Procedure of SAS software (SAS Institute, Inc., Cary, NC, United States). A standard analysis of variance (ANOVA) of the aggressiveness indices on each of the four hosts was performed using the SAS software (SAS Institute, Inc., Cary, NC, United States) to test the effect of the host cultivar, the field and the genetic group on strain aggressiveness. Moreover, the mean-values of aggressiveness indices (i.e., Ag-K, Ag-T, Ag-P, Ag-Pl), of initial lesion expansion rate (α_Na_) and of final lesion area (Na) were compared using the Student’s *t*-test. Means values were considered as different for a probability under the threshold of 0.01.

## Results

### Genetic Diversity

A total of 222 monoconidial strains of *C. gloeosporioides* were isolated from three host cultivars (Figure 1). The AFLP analysis generated 89 clearly reproducible bands. Among the 89 clearly reproducible markers, 23 were polymorphic, independent and had a frequency between 0.05 and 0.95, i.e., non-rare alleles. All the markers were amplified from several isolates originating from at least three different fields. Among the 222 isolates, 197 haplotypes were identified, which correlated with high *R* ratios (i.e., modified *G*/*N* ratio, where the number of identical multilocus genotypes, *G*, is divided by the number of individuals, *N*) recorded for the entire dataset, 0.89, and in each field-population, from 0.83 to 0.93 (Table 1). Sixteen haplotypes were found in two, three or four copies, but the few isolates sharing the same haplotype were always recovered from a unique field (Supplementary Figure S3). Nei (1987) gene diversities were all superior to 0.20 whether they were calculated on field sample or on the entire dataset (Table 1).

**Table 1:** Genetic diversity indexes within the pooled sample of *Colletotrichum gloeosporioides* and samples from each field.

Origin of the isolates		# isolates	# haplotypes	# loci^(a)^	H n.b.^(b)^	R^(c)^
Field	Host *cv*					
All	All	222	197	23	0.39 *(0.12)*	0.89
Kab_A_	Kabusah	25	21	22	0.23 *(0.19)*	0.83
Kab_B_	Kabusah	40	36	18	0.25 *(0.16)*	0.90
Pac_c_	Pacala	35	32	22	0.20 *(0.18)*	0.91
Pac_D_	Pacala	40	36	21	0.33 *(0.15)*	0.90
Tah_A_	Tahiti	41	34	22	0.22 *(0.18)*	0.83
Tah_B_	Tahiti	41	38	23	0.31 *(0.17)*	0.93

*^(a)^Number of polymorphic loci out of the 86 loci. ^(b)^Non-biased Nei (1987) diversity; standard errors are specified in brackets. ^(c)^R, the debiased G/N index calculated as (G−1)/(N−1) with G, the number of identical multilocus genotypes and N, the number of individuals.*

### Population Structure

**FIGURE 2:**
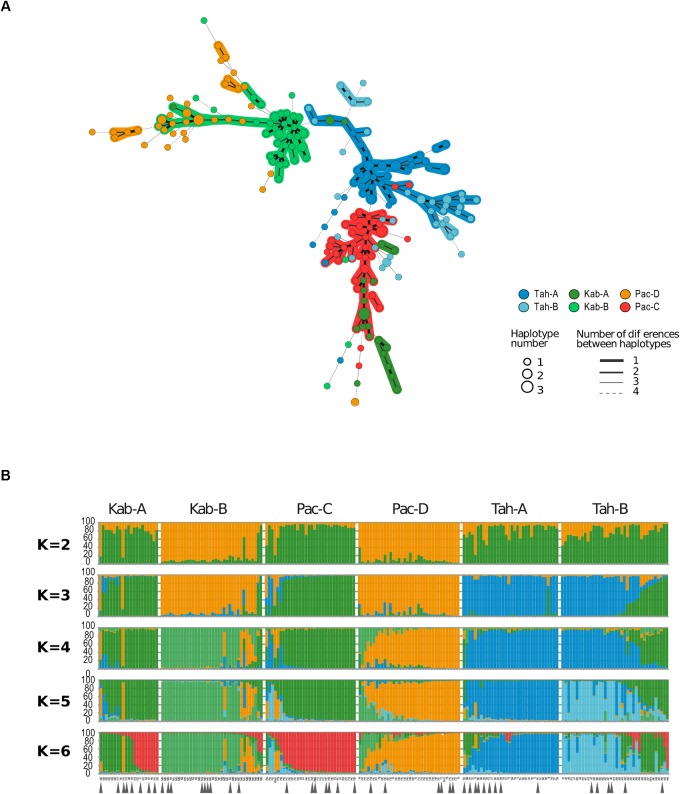
Genetic structure of the 222 *Colletotrichum gloeosporioides* strains sampled on six fields of three different cultivars. **(A)** The minimum-spanning tree based on ALFP markers was constructed using Bionumerics 5.1. Haplotypes collected on *cv* Tahiti are coded in blue, on *cv* Kabusah in green and on *cv* Pacala in Red (bottom right). The size of the circles corresponds to the number of replicates of the same haplotype (bottom right). Numbers of differences between haplotypes are coded by the type of line joining two circles (bottom right). **(B)** Structure software output based on 23 AFLP markers with the number of genetic groups (K) ranging from 2 to 6. Kab, Tah, Pac, respectively, stands for the host, *Dioscorea alata* cultivars Kabusah, Tahiti and Pacala. The letters A, B, C, D stand for the four geographical origins (see Figure 1). Gray arrows indicate the strains randomly chosen for the pathogenicity tests.

The population structure analysis with no *a priori*, displayed in the Minimum Spanning Tree (Figure 2A) was concordant with the structure analysis using both the Structure software (Figure 2B) and the DAPC—adegene package (Supplementary Figures S4–S6). According to Evanno’s method, the clone-corrected sample (197 isolates) was most likely divided into three genetic groups (Supplementary Figure S2). The group 1 (blue in Figure 2B, *k* = 3) was composed of more than 98% of strains isolated from yam *cv* Tahiti. This “Tahiti-exclusive” genetic group contained 78.8% of the strains collected on *cv* Tahiti, including 97% of the field Tah_A_ sample and 61% of the field Tah_B_ sample. For the groups 2 and 3 (green and orange in Figure 2B, *k* = 3) the clustering did not correspond to one host *cv* nor to one geographical location. The group 2 (green in Figure 2B, *k* = 3) was composed of isolates from the fields Pac_C_ (50%), Kab_A_ (33%), and Tah_B_ (13.5%). The group 3 (orange in Figure 2B, *k* = 3) contained the entire Pac_D_ sample and 91.6% of Kab_B_ sample, which represented 93% of the total of strains contained in this genetic group. The genetic grouping was more complex for the group 2. Pac_C_ strains belonged to one genetic group only, but Kab_A_ strains belonged to two genetic groups, one common with strains originated from Tah_B_ and one with strains from Pac_C_.

The computed pairwise-Fst proved that populations from different fields were significantly differentiated, whatever the host cultivar, *cv*, and the field (Table 2). The pairwise-Fst values were clearly consistent with the genetic clustering given by the analysis with *K* = 6. Indeed, the “Tahiti-private” group and the group 3, respectively, split into two genetic groups corresponding to distinct field of origin (i.e., Tah_A_ vs Tah_B_; Pac_D_ vs Kab_B_). The lowest Fst-value (0.161) was observed between the fields Kab_A_ (host *cv* Kabusah) and Pac_C_ (host *cv* Pacala) distant of 1.3 km, whereas higher Fst-values (0.406 and 0.404) were observed between two contiguous fields (Kab_A_ and Tah_A_) and between the two *cv* Pacala fields (Pac_C_ and Pac_D_).

**Table 2:** Pairwise-Fst among the six *Colletotrichum gloeosporioides* populations (222 isolates) collected from Yam (*Dioscorea alata*) fields in Morne-à-l’eau (French West Indies, Guadeloupe), estimated with 23 polymorphic AFLP-markers.

Origin of the strains							
Host *cv*	Field	Kab_A_	Kab_B_	Pac_c_	Pac_D_	Tah_A_	Tah_B_
Kabusah	Kab_A_	–	*0.0081*	*0.0035*	*0.0090*	*0.0054*	*0.0170*
Kabusah	Kab_B_	0.364^∗^	–	*0.0100*	*0.0017*	*0.0000*	*0.0046*
Pacala	Pac_c_	0.161^∗^	0.463^∗^	–	*0.0076*	*0.0002*	*0.0250*
Pacala	Pac_D_	0.346^∗^	0.282^∗^	0.404^∗^	–	*0.0004*	*0.0045*
Tahiti	Tah_A_	0.406^∗^	0.379^∗^	0.244^∗^	0.436^∗^	–	*0.0003*
Tahiti	Tah_B_	0.364^∗^	0.341^∗^	0.291^∗^	0.282^∗^	0.243^∗^	–

*Below the diagonal: Pairwise-Fst were calculated following the Weir and Cockerham (1984) indications. ^∗^indicates significant Fst (p < 0.001) after 1,000 permutations. Above the diagonal: Exact p-values of the exact test of sample differentiation based on haplotype frequencies with Markov chain length of 10,000 steps (Raymond and Rousset, 1995; Goudet et al., 1996).*

Altogether, the populations sampled on the cultivar Tahiti tend to form a “Tahiti-specific’ group, the populations from the fields Pac-D and Kab-B form two clearly distinct genetic groups. The sample from the Kab-A field does not form a uniform genetic group, as to a lesser extent, the sample from the fields Pac-C and Tah-B.

### Indices of Association

All indices of association values (*r*_D_) calculated were low (*r*_D_-values ranging from 0.003 to 0.103) but with significant departure to 0 for all populations except cluster 1 (Table 3).

**Table 3:** Standardized index of association within *Colletotrichum gloeosporioides* populations.

Population	*r*D^(a)^	*r*D^∗(b)^
All	0.031^∗∗^	
Kab_A_	0.056^∗∗^	
Kab_B_	0.103^∗∗^	
Pac_c_	0.032^∗∗^	
Pac_D_	0.036^∗∗^	
Tah_A_	0.049^∗∗^	
Tah_B_	0.029^∗∗^	
All		0.024^∗∗∗^
Cluster 1		0.016
Cluster 2		0.03^∗∗^
Cluster 3		0.028^∗∗∗^

*^(a)^rD: standardized index of association calculated on the clone-corrected data set using 23 ploymorphic markers, i.e., with the haplotypes (Haubold et al., 1998; Agapow and Burt, 2001). ^(b)^rD^∗^: standardized index of association calculated on the clone-corrected data set using 16 polymorphic markers where loci had no significant LD with each others; ^∗∗^indicates significant values with p-values below 0.01 after 1,000 random resampling, ^∗∗∗^indicates significant values with p-values below 0.001 after 1,000 random resampling.*

### Strain Aggressiveness Distribution

**FIGURE 3:**
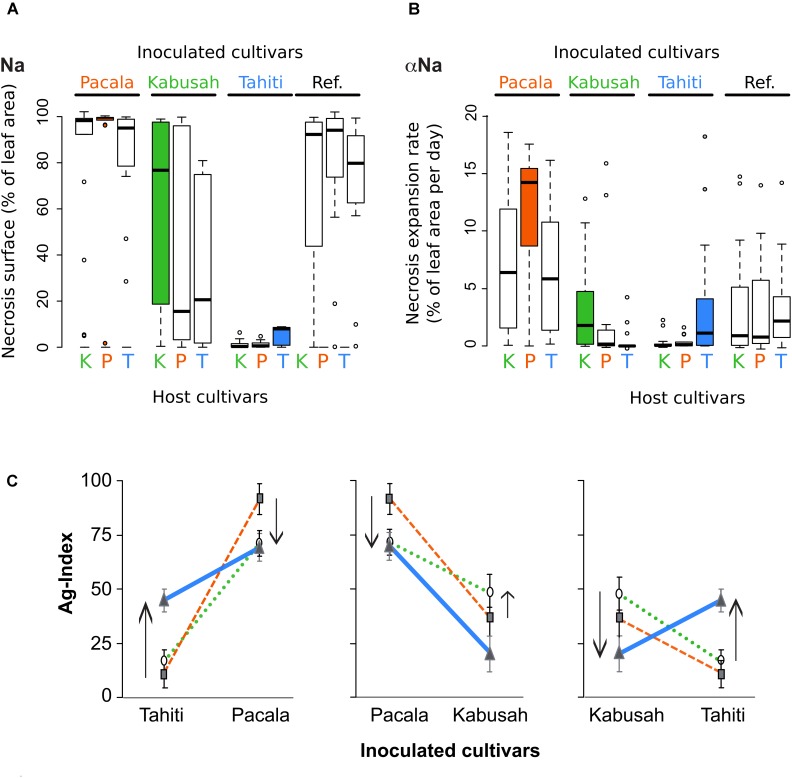
*C. gloeosporioides* local adaptation to its host resistance. A subsample of 45 *C. gloeosporioides* strains isolated from three *D. alata* host cultivars Kabusah (K, moderate susceptibility), Pacala (P, high susceptibility) and Tahiti (T, low susceptibility) were cross-inoculated on these cultivars and on a cultivar of reference (Plimbite, Ref.). **(A)** Boxplot for the necrosis area, Na. **(B)** Boxplot for the initial (i.e., day 0–day 6) necrosis expansion rate, α_NA_ were obtained for each cross-inoculation using R-software. **(C)** Pairwise comparison of the strain aggressiveness inoculated on their local and foreign hosts. Aggressiveness of the strains isolated from *cv* Kabusah are, respectively, represented with black circles (green dotted lines), from *cv* Pacala in black square (red dashed lines) and from *cv* Tahiti in gray triangles (blue lines). Arrows underline the loss or the gain of mean aggressiveness in comparison with the one of strains isolated on *cv* Pacala, the oldest and susceptible cultivar.

The diversity for strain aggressiveness was investigated on a 45-strain subsample. These strains belong to the main genetic groups described above (Figure 3 and Supplementary Figure S7). No symptom nor chlorosis appeared on control leaves 11 days after inoculation. We detected an effect of the genetic group (cluster) on strain aggressiveness on the cultivar Tahiti but none on the cultivars Kabusah, Pacala and Plimbite (Table 4). Besides, strain aggressiveness on yam *cv* Plimbite (taken as the reference for susceptibility) and on yam *cv* Kabusah (moderate susceptibility) did not depend on the host nor on the field of origin (Table 4). Nevertheless, the Student’s *t*-test showed that strains collected from *cv* Kabusah were more aggressive on their host *cv* Kabusah (Ag-index mean-value of 47.8) than the strains sampled from *cv* Tahiti (Ag-index mean-value of 20.3). More strikingly, strain aggressiveness, respectively, on the yam *cv* Tahiti (low susceptibility) and *cv* Pacala (high susceptibility), significantly depended on the host cultivar and the field of origin (Table 4). The mean aggressiveness on *cv* Tahiti was higher for the strains collected on this cultivar (mean Ag-index value of 44.7) than the strains from other host yam *cv* (mean Ag-index values of 16.6 and 11.7). In the same way, the strains collected on *cv* Pacala displayed the highest level of aggressiveness on *cv* Pacala than the other strains (Table 4). Finally, the effect of the host cultivar on the values for the initial lesion expansion rates (α_Na_; Figure 3) and the typical lesion area on day 11 (Na; Figure 3) was similar to the effect observed on the Ag-index (aggressiveness). The initial lesion expansion rate seemed critical to explain the adaptive evolution of the aggressiveness.

**Table 4:** Aggressiveness indices (Ag) of *Colletotrichum gloeosporioides* populations from six *D. alata* yam fields tested on four yam cultivars: Kabusah, Pacala, Tahiti and Plimbite (the cultivar of reference for the susceptibility to anthracnose).

	*N*	Aggressiveness (Ag-index) estimated on four									*D.alata*	cultivars
Levels		*cv Kabusah*			*cv Pacala*			*cv Tahiti*			*cv Plimbite* (ref)	
host *cv* Kabusah	17	47.8	*(7.5)*	a	71.1	*(6)*	b	16.6	*(5.2)*	b	51.6	*(7.2)* a
host *cv* Pacala	13	36.4	*(9)*	ab	91.5	*(7.1)*	a	11.7	*(6.1)*	b	56.6	*(8.6)* a
host *cv* Tahiti	15	20.3	*(8.3)*	b	69.3	*(6.4)*	b	44.7	*(5.5)*	a	54	*(7.7)* a
Kab_A_ – field	8	41.4	*(5.5)*	ab	59.9	*(8.2)*	ab	12.1	*(7.5)*	b	43.4	*(10.4)* a
Kab_B_ – field	9	53.4	*(6.3)*	a	81.1	*(7.7)*	ab	20.7	*(7)*	b	59	*(9.8)* a
Pac_c_ – field	7	48	*(5.9)*	a	90	*(8.2)*	a	13.5	*(7.5)*	b	65.9	*(10.4)* a
Pac_D_ – field	6	13.3	*(5.7)*	b	94.5	*(11.6)*	a	8	*(10.5)*	b	38	*(14.7)* a
Tah_A_ – field	9	25.8	*(10.8)*	ab	58.8	*(7.7)*	b	37.1	*(7)*	a	59.7	*(9.8)* a
Tah_B_ – field	6	13	*(12.4)*	b	85	*(9.5)*	ab	56.2	*(8.6)*	a	45.5	*(12)* a
Genetic cluster 1	13	23.3	*(9.3)*	a	66.2	*(6.9)*	a	43.4	*(6.3)*	a	53.3	*(8.3)* a
Genetic cluster 2	15	42.4	*(5.2)*	a	73.2	*(6.5)*	ab	17.5	*(5.9)*	b	54	*(7.7)* a
Genetic cluster 3	16	38.6	*(6.1)*	a	86.8	*(6.3*	a	16.8	*(5.7)*	b	54	*(7.5)* a
prob F, effect = host cultivar		0.061			0.048^∗^			0.000^∗∗∗^			0.908	
prob F, effect = field of origin		0.075			0.019^∗^			0.002^∗∗∗^			0.472	
prob F, effect = genetic group (cluster)		0.294			0.088			0.005^∗∗∗^			0.998	

*Fields KabA/Tah_A_, and KabB/Tah_B_ were contiguous (see Figure 1). a, b stands for the groups of significant differences of aggressiveness tested using Student’s t-test with a p-value p < 0.01. Tests were performed considering separately each of the four inoculated cultivars and each of the fungal population grouping levels, host cv, field and genetic cluster. ^∗^,^∗∗∗^, respectively, indicates significant values with p-values below 0.05 and 0.001. Standard errors are specified in brackets.*

### Patterns of Strain Adaptation to Its Host Cultivar

The pairwise comparison of strain aggressiveness (Ag-indices) on the three host *cv* populations on the same inoculated *cv* (Table 4 and Figure 3) revealed clear pattern of local adaptation on *cv* Tahiti and *cv* Kabusah with high differences in mean aggressiveness values. Local adaptation to the host *cv* Pacala (highly susceptible) was also shown, however, the mean aggressiveness values were all higher on *cv* Pacala than on the other inoculated cultivars which could be seen as a general adaptation to this cultivar. The patterns of local adaptation to the host cultivar were reinforced by the absence of any pattern of differential adaptation of the strains to the cultivar of reference, *cv* Plimbite. Finally, the pattern of *C. gloeosporioides* adaptation to its host cultivar, especially to the cultivar Tahiti, was similar to the pattern expected for locally adapted pathogen populations.

## Discussion

Our purpose was to better understand how and in which proportions the migration, recombination and yam quantitative resistance influence the *C. gloeosporioides* evolution, and more particularly the evolution of *C. gloeosporioides* pathogenicity. Does the adaptive pattern of *C. gloeosporioides* correspond to a general adaptation to the most common host cultivar, or to a highly fragmented pathogen population resulting from the adaptation to each cultivar subpopulation (i.e., local adaptation; Gandon et al., 1996; Gandon and Van Zandt, 1998)? Furthermore, can the history of cultivars’ succession in Guadeloupe be reflected by the recorded fungal pathogenicity distribution?

### *Colletotrichum gloeosporioides* Diversity and Sexual Reproduction Imprint

The AFLP analysis revealed high genetic diversity. Such levels for neutral molecular diversity were previously recorded for *C. gloeosporioides* collected from different yam hosts in three agro-ecological areas of Nigeria (Abang et al., 2006). The indirect evaluation of the reproductive mode, that is the standardized index of multilocus LD, was in accordance with a significant departure from total panmixia, but not reaching complete clonality. Our results thus revealed significant the impact of clonal reproduction on *C. gloeosporioides*’s evolution, which is in concordance with the role of conidia dispersal in the epidemic expansion and the presence of quiescent mycelia in tubers. However, the influence of recombination (sexuality) was not entirely excluded. Besides previous studies reported the presence of sexual reproductive structures of *C. gloeosporioides* on dead yam leaves at the end of the crop season (Toribio and Jacqua, 1978; Toribio et al., 1980; Degras et al., 1984; Abang et al., 2001, 2006). Therefore, it seems parsimonious to consider that recombination explains the high haplotype diversities observed in every subset. Altogether, the influence of sexual reproduction and the high levels of genetic diversity seem to be general traits in *C. gloeosporioides*’s biology as it was previously suggested for *C. gloeosporioides* populations damaging yams (Mignouna et al., 2002; Abang et al., 2005, 2006) and damaging strawberry (Xiao et al., 2004). To conclude, even if *C. gloeosporioides*, pathogenic on *D. alata*, undergoes weak recombination between two crop seasons, sexuality likely confers this fungus a great opportunity to evolve.

### *Colletotrichum gloeosporioides* Migration Rate

The haplotype distribution within one population gives insights into its dispersal potential (McDonald, 1997). In the present study, the AFLP genotyping of the haploid fungus *C. gloeosporioides* revealed a strong population structure: each field sample can be considered as one fungal population. The number of genetic groups revealed with the no *a priori* analyses and the high Fst-values recorded between field populations, whatever their location or host cultivar, suggest a weak influence of *C. gloeosporioides* migration between the fields studied during the crop season. Indeed, no haplotypes were shared between fields. Moreover, the strains from adjoining fields (Kab_A_-Tah_A_ and Kab_B_-Tah_B_; Figure 1 and (Supplementary Figure S3) belonged to different genetic groups, whereas individuals from the field Pac_C_ (Pacala) were genetically close to some of the individuals from the field Kab_A_ (Kabusah). Furthermore, out of the 18 multicopy haplotypes, only seven were sampled from neighboring sampling positions whereas 11 were sampled from dispersed sampling positions within the same field (Supplementary Figure S3). In other words, for the fungal populations studied here, the dispersal by splashing during the crop season occurred but had a minor impact at a local scale (i.e., not beyond few meters).

In fact, the population structure recorded here (high diversity and strong structure) corresponds to the one expected for a primary inoculum. It is also consistent with the correlation found between the presence of *C. gloeosporioides* mycelia in the yam tubers planted and the subsequent leaf anthracnose severity (Green and Simons, 1994). Ultimately, as farmers usually divide yam tubers into pieces (seeds) and save them for the next crop season, when a tuber is infected with one isolate, this isolate will be randomly dispersed within a field where seeds are planted. This fully explains the genetic diversity we observed within and between fungal field-populations. More importantly, the spatial distribution of the haplotypes seemed more likely related to seeds distribution than to fungus dispersal by splashing.

Finally, if dead stems and leaves left on the soil after the previous crop season are a potential source of primary inoculum (Ripoche et al., 2007), the impact of such event cannot be detected in this study, as for all the fields the previous crop was sugar cane. Although no strong influence of migration was detected in this study, the pathogen migration can occur between fields of severely damaged plants (Degras et al., 1984; Green, 1994).

### Colletotrichum Gloeosporioides Local Adaptive Response to Yam Resistances

Previous works already reported the ability of *C. gloeosporioides* to overcome host resistance (Miles and Lascano, 1997; Chakraborty et al., 1999; Abang et al., 2001; Abang, 2003) or to develop resistance to fungicides (Bayart and Pallas, 1994). The last point of our investigations focused on the adaptation of the field-structured populations to their host quantitative resistance. Are *C. gloeosporioides* field populations adapted to every host cultivar (general adaptation) or are they specifically adapted to their host cultivar?

We can also draw a parallel between the patterns of adaptation recorded and the “age” and intensity of each cultivar’s production in Guadeloupe. As a matter of fact, the cultivar Pacala was the most widespread in Guadeloupe until the end of the 1960s, when its resistance to anthracnose was overcome. Despite its susceptibility to anthracnose, *cv* Pacala is still one of the most planted cultivars in Guadeloupe (Penet et al., 2016). The *cv* Plimbite, introduced during the 1970s, was widely cultivated in Guadeloupe but its prevalence decayed when its resistance was overcome in the early 1990s (Ano et al., 2002). The water yam *cv* Kabusah was the last cultivar introduced at the beginning of the 1990s and has been widely cultivated ever since. Finally, *cv* Tahiti has been cultivated from the 1960s but its prevalence increased in the 1990s.

The patterns of adaptation described in the present study are concordant with a general adaptation to the formerly highly prevalent hosts (*cv* Pacala and *cv* Plimbite) and a local adaptation (i.e., diversifying selection) to the cultivars which became abundant more recently (*cv* Kabusah and *cv* Tahiti; Table 4 and Figure 3). Surprisingly a pattern of local adaptation to the old but still widely planted *cv* Pacala was observed despite a strong general adaptation pattern, revealing an ongoing selective pressure of *cv* Pacala on *C. gloeosporioides* populations.

In our sample, strain virulence (not null aggressiveness) on the four yam cultivars prevailed. It is unclear whether this corresponds to an accumulation of virulences within *C. gloeosporioides* populations or to a core virulence component fixed in all populations. The first hypothesis seems more likely as a former survey on Brazilian *C. gloeosporioides* populations infecting *Stylosanthes* showed an increase of fungal race complexity within few years (Chakraborty et al., 2002). The overall virulence of the studied strains reveals that the recorded adaptation for aggressiveness does not depend on the virulence combination. The adaption of *C. gloeosporioides* to its yam host is quantitative. Besides, we did not find any correlation between strain aggressiveness (Ag-values) against the Kabusah, Tahiti or Pacala cultivars (Supplementary Figure S8). This indicates a certain level of specificity in the *C. gloeosporioides*–*D. alata* interaction, even if it seems minor compared to the quantitative aspect. The evolutionary potential of the *C. gloeosporioides* populations pathogenic on *D. alata* reinforces the necessity to better understand the aggressiveness evolution in time and space in order to efficiently predict the durability of *D. alata* resistance to anthracnose. Further investigations of the in-field population dynamics are needed to evaluate the potential of aggressiveness evolution during one crop season. Finally, as previously mentioned, the yam tuber infection can be crucial for the parasite long-distance dispersal and its survival between crop seasons. The capacity of *C. gloeosporioides* to infect and survive in its host tuber is clearly a determinant aspect of its own fitness thus its evolutionary potential. The pattern of genetic diversity revealed by our study underlines the need to improve the control for seed quality at all cost.

## Author Contributions

LF, GJ, and CN contributed to conception and design of the study. LF analyzed the results and wrote the first draft of the manuscript. CN supervised the work. All authors contributed to manuscript revision, read and approved the submitted version.

## Conflict of Interest Statement

The authors declare that the research was conducted in the absence of any commercial or financial relationships that could be construed as a potential conflict of interest.

## Supplementary Material

FIGURE S1 Power of the number of markers used to detect genotypes and measure diversity.

FIGURE S2 Evanno’s Delta *K* test.

FIGURE S3 Detailed distribution of haplotypes at the field scale. The relative positions where the haplotypes where sampled are specified. The genetic groups obtained in the Structure analysis with *K* = 3, are color coded in orange, green, and blue. Strains belonging to several genetic groups are specified using dashed color code. The letters T, A, K, M, P, F correspond to the strains, respectively, originating from the fields specified in Figure 1, Tah-A, Tah-B, Kab-A, Kab-B, Pac-C, Pac-D. The strains found to be the same haplotype are specified using black boxes, the names of the duplicates are given with the letters a, b, c. No haplotypes were shared between fields.

FIGURE S4 Principal Component Analysis of the 199 haplotypes. PCA was performed using the adegenet-DAPC software (Jombart and Ahmed, 2011). **(A)** The PCA shows the dispersion of the 197 haplotypes (23 independent AFLP markers). Colors represent the cultivar of origin: Tahiti, Kabusah, Pacala.

FIGURE S5 Principal Component Analysis of the 199 haplotypes. PCA was performed using the adegenet software (Jombart and Ahmed, 2011). The PCA shows the dispersion of the 197 haplotypes (23 independent AFLP markers) when the number of groups is 6 or 3. For each group defined by the DAPC analysis, the number of haplotypes sampled in each field specified in Figure 1 (i.e., Tah-A, Tah-B, Kab-A, Kab-B, Pac-C, Pac-D) are specified in the tables.

FIGURE S6 Principal Component Analysis of the 199 haplotypes. DPCA was performed using the adegenet software (Jombart and Ahmed, 2011). The DPCA shows the dispersion of the 197 haplotypes (23 independent AFLP markers) when the number of groups is, respectively, 4, 5, 7, and 8.

FIGURE S7 Structure groups for the strains used in the pathogenicity tests. Structure software output based on 23 AFLP markers with the number of genetic groups (K) ranging from 2 to 6. Kab, Tah, Pac, respectively, stands for the host, *Dioscorea alata* cultivars Kabusah, Tahiti, and Pacala. The letters A, B, C, D stand for the four geographical origins (see Figure 1).

FIGURE S8 Pairwise correlation between *C. gloeosporioides* aggressiveness (Ag-Index values) against four *D. alata* cultivars. The psych package (Revelle, 2018) was used to plot and test the pairwise regressions of the 45 *C. gloeosporioides* strains’ aggressiveness recorded on four different *D. alata* cultivars, *cv* Kabusah, Pacala, Tahiti, and Plimbite. Below the diagonal: pairwise scatter plots of Ag-indexes, respectively, assessed on *cv* Kabusah (KAB), Pacala (PAC), Tahiti (TAH), and Plimbite (PLI). Diagonal: the histogram and lowess locally fit regression line of the Ag-indexes assessed on each cultivar, respectively. The *x* axis in each scatter plot represents the column Ag-indexes, the *y* axis the row Ag-indexes. Above the diagonal: the Pearson correlation values. The significances of single correlation were tested with multiple comparisons adjustment using the Holm correction and are specified as ^∗∗∗^ for *p* ≤ 0.001 and ^∗∗^ for *p* ≤ 0.01.

TABLE S1 Main characteristics of the six Dioscorea alata fields located in the center of Guadeloupe (Morne-à-l’eau). **(A)** Sugar-cane (sc) and pasture (p); TahA/B and KabA/B refers to the adjoining fields described in Figure 1; **(B)** Farm indicates that the tuberpieces (i.e., seeds) planted were harvested during the previous crop-season in the same farm. Imported, means that tuber-pieces planted were obtained from other farms or, more likely imported from Costa Rica.
