# Supplementary material for: Adaptation of a Fungal Pathogen to Host Quantitative Resistance
Source: Front Plant Sci. 2018 Oct 31;9:1554. doi: 10.3389/fpls.2018.01554 (PMC6220312; doi:10.3389/fpls.2018.01554)

Table S1 Main characteristics of the six *Dioscorea alata* fields located in the center of Guadeloupe (Morne-à-l'eau).

Origin of the strains		collection date	host sowing date	previous culture [a]	adjoining cultures [a]	seeds' origins [b]	inter-rows (cm)	inter-plants (cm)
host cultivar	field							
Kabusah	Kab_A	18 dec. 01	June	sc	sc + Tah_A	farm	150	20
Kabusah	Kab_B	15 jan. 02	Aug/ Sept	sc	sc + p + Tah_B	imported	150	30
Pacala	Pac_C	11 dec. 01	May/ June	sc	sc + cv *Kabusah*	farm	160	20-25
Pacala	Pac_D	08 jan. 02	Aug/ Sept	sc	sc + p	farm	160	20-25
Tahiti	Tah_A	14 dec. 01	July	sc	sc + Kab_A	farm	150	20
Tahiti	Tah_B	24 jan. 02	June	sc	sc + kab_B	farm	150	30

a: sugar-cane (sc) and pasture (p); $Tah_{A/B}$ and $Kab_{A/B}$ refers to the adjoining fields described in Fig.1; **b**: farm indicates that the tuber-pieces (i.e. seeds) planted were harvested during the previous crop-season in the same farm. Imported, means that tuber-pieces planted were obtained from other farms or, more likely imported from Costa Rica.

Fig. S1

Loci Sampled	Mean # Genotype	Std Error	Mean_Div	Std_Er(Div)
1	2.00	0.000000	0.38	0.011284
2	4.00	0.000000	0.62	0.010128
3	7.75	0.050000	0.76	0.006463
4	14.45	0.143812	0.86	0.004968
5	23.39	0.319689	0.90	0.003683
6	35.73	0.510071	0.93	0.002198
7	50.78	0.727883	0.95	0.001785
8	66.57	0.879193	0.97	0.001001
9	82.91	1.024950	0.98	0.000685
10	98.90	0.961113	0.98	0.000498
11	110.38	1.010230	0.99	0.000380
12	122.87	0.935782	0.99	0.000305
13	134.63	1.022740	0.99	0.000247
14	146.15	0.996496	1.00	0.000188
15	152.65	0.898526	1.00	0.000144
16	159.31	0.944382	1.00	0.000138
17	168.57	0.809945	1.00	0.000094
18	174.14	0.651187	1.00	0.000067
19	180.39	0.601328	1.00	0.000052
20	185.32	0.511461	1.00	0.000039
21	189.12	0.406582	1.00	0.000025
22	192.82	0.276844	1.00	0.000015
23	196.00	0.000000	1.00	0.000000

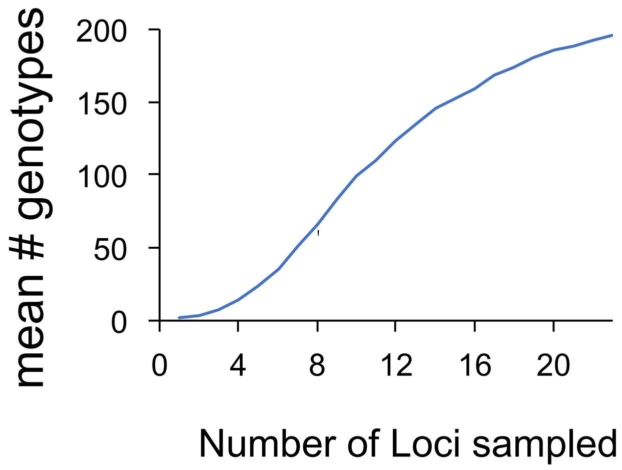

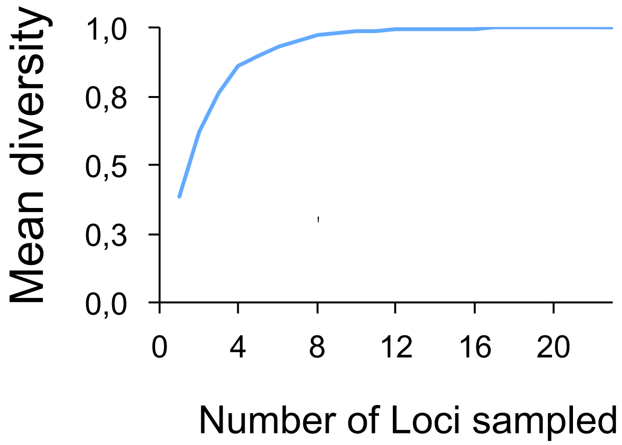

Fig. S2

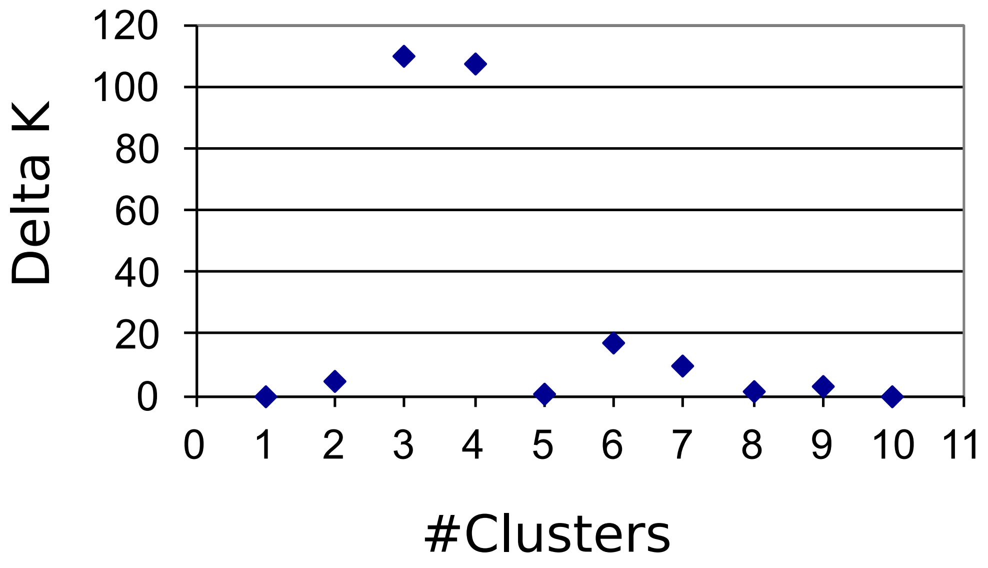

Fig. S3

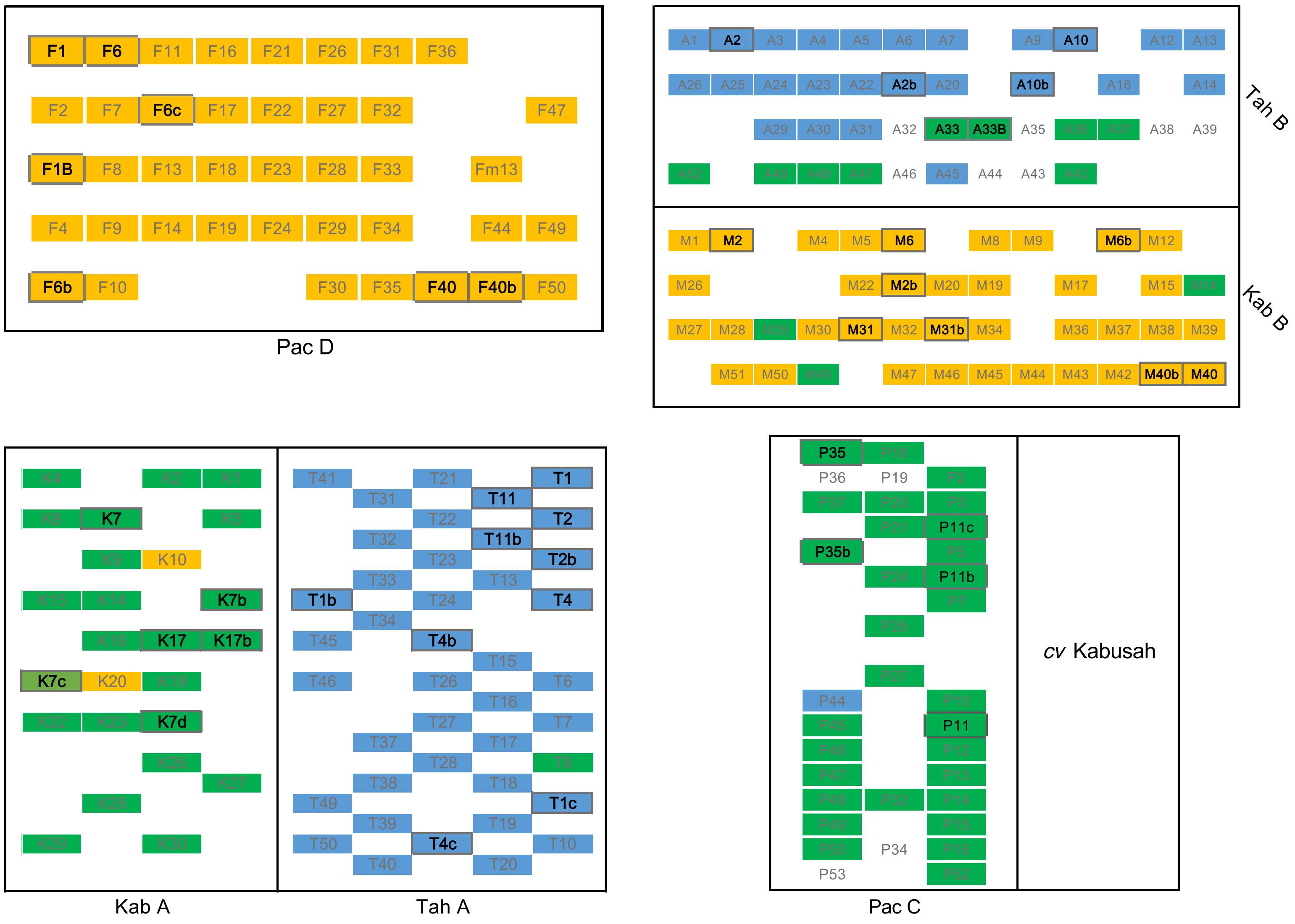
Pac D
F1 F6 F6c F1B F6b F40 F40b
Tah B
A2 A10 A2b A10b A33 A33B
A32 A35 A38 A39 A46 A44 A43
Kab B
M2 M6 M6b M2b M31 M31b M40b M40
Kab A
K7 K7b K17 K17b K7c K7d
Tah A
T1 T11 T2 T11b T2b T1b T4 T4b T1c T4c
Pac C
P35 P36 P19 P11c P35b P11b P11 P34 P53
cv Kabusah

Fig. S4

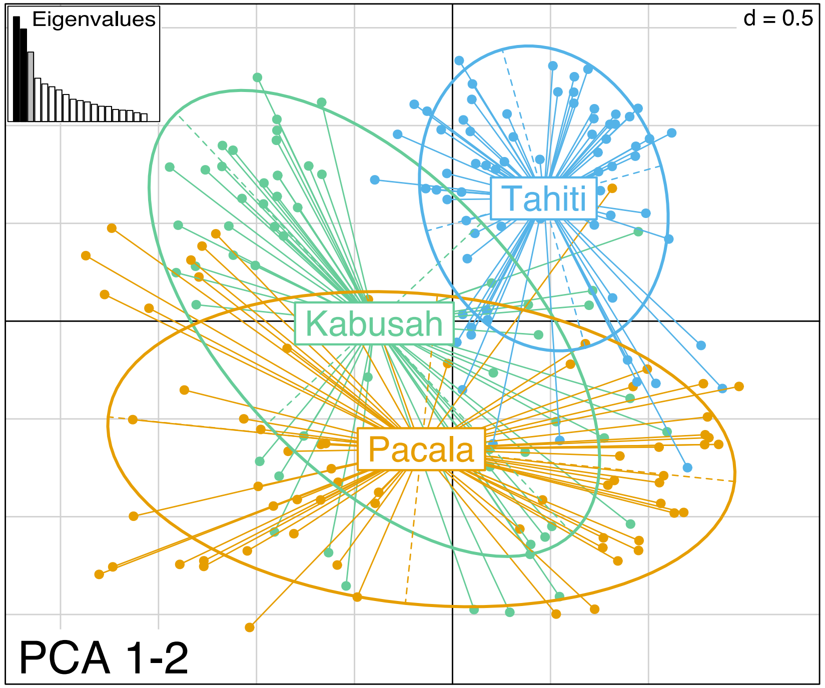

Fig. S5

PCA with 6 groups

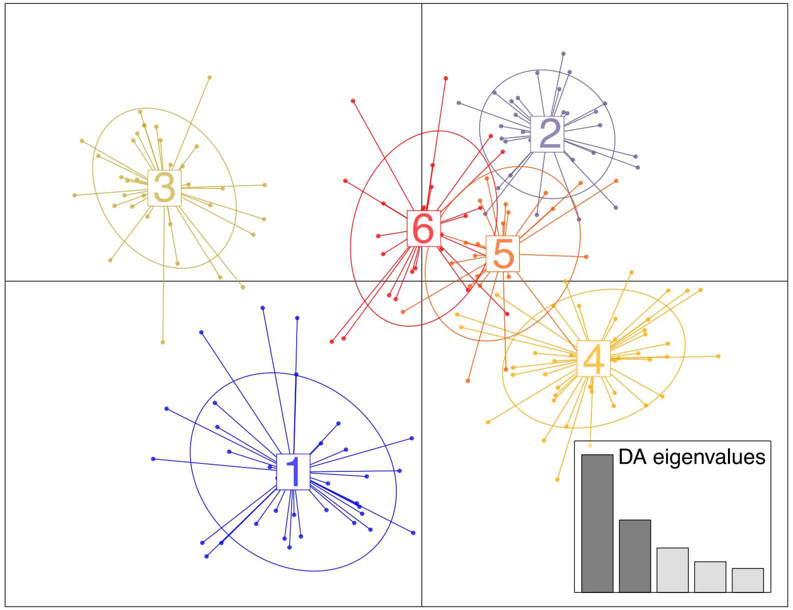

Fields	Group1	Group2	Group3	Group4	Group5	Group6
Kab-A	1	0	0	9	0	11
Kab-B	4	0	29	2	0	1
Pac-C	1	0	0	28	2	1
Pac-D	28	0	7	0	0	1
Tah-A	0	29	0	1	0	4
Tah-B	0	2	0	5	22	9

PCA with 3 groups

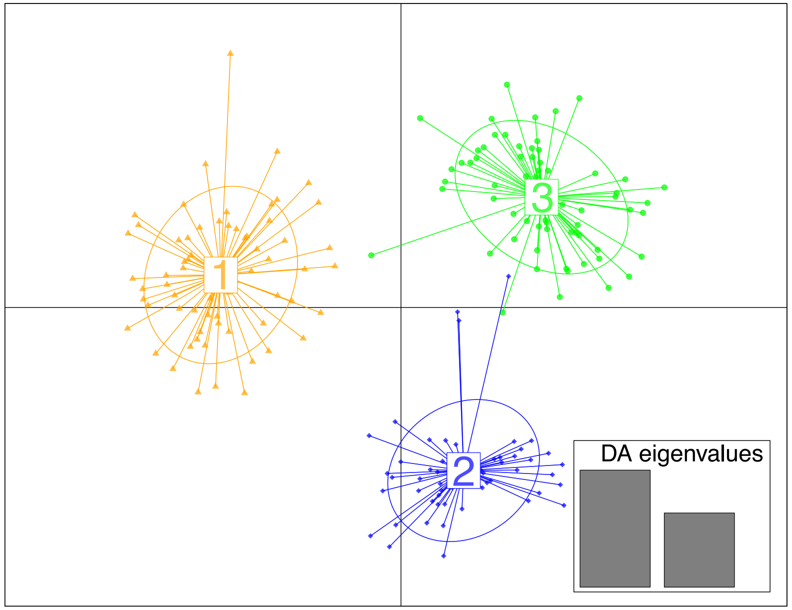

Fields	Group1	Group2	Group3
Kab-A	2	19	0
Kab-B	33	3	0
Pac-C	1	28	3
Pac-D	36	0	0
Tah-A	0	1	33
Tah-B	0	15	23

Fig. S6

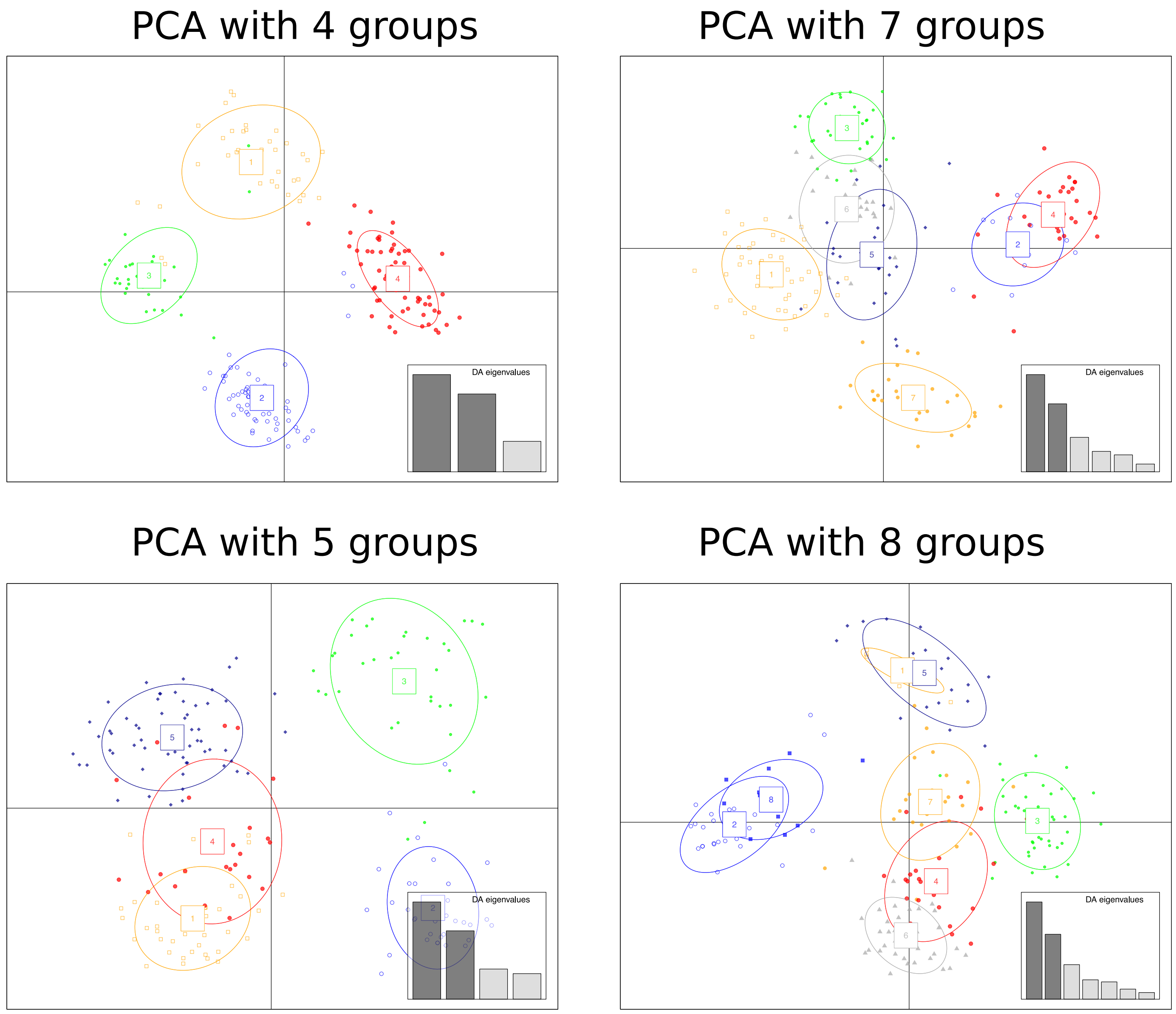
PCA with 4 groups
PCA with 7 groups
PCA with 5 groups
PCA with 8 groups
DA eigenvalues
DA eigenvalues
DA eigenvalues
DA eigenvalues
1
2
3
4
5
6
7
8

Fig S7

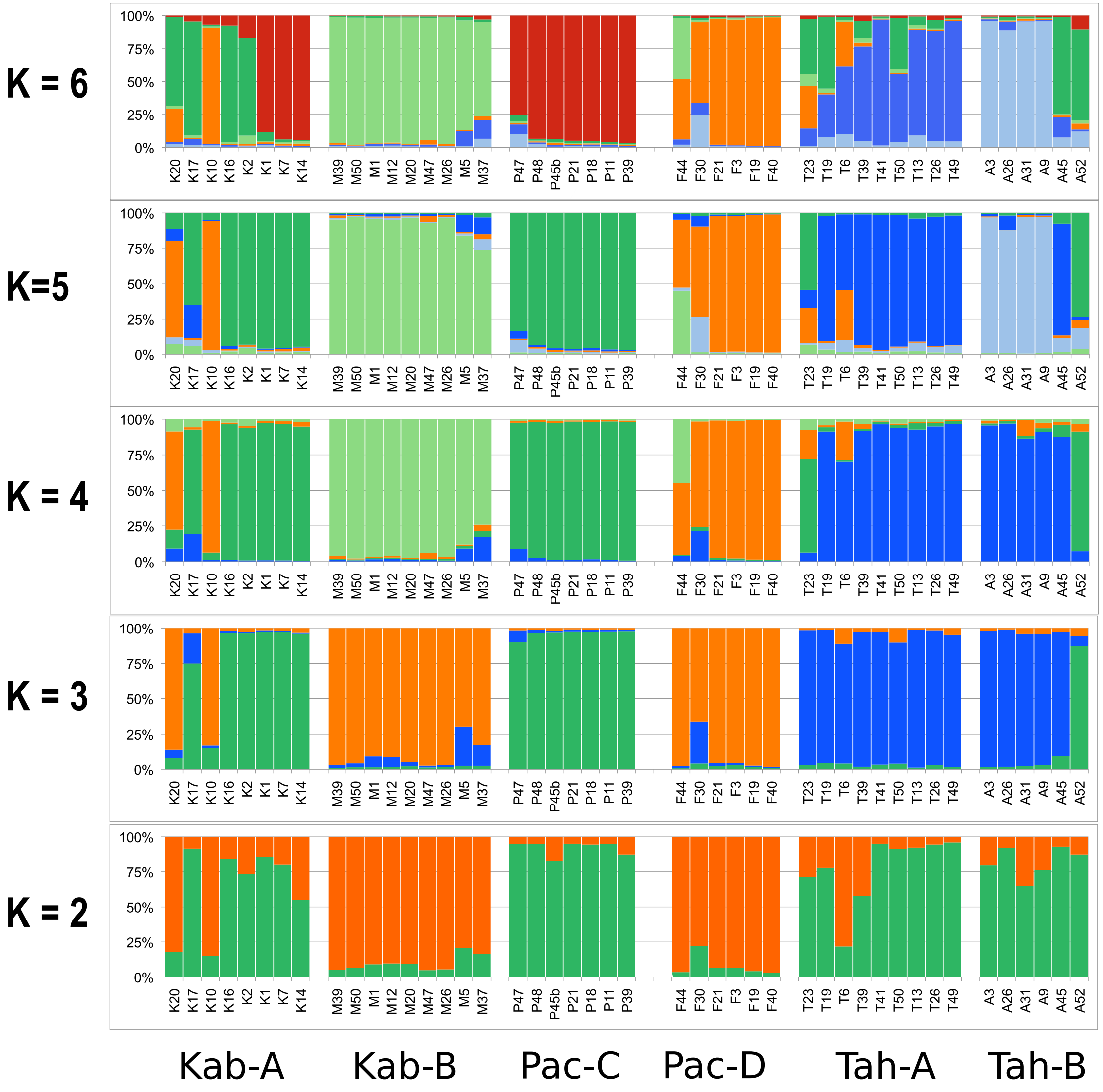

K = 6
K=5
K = 4
K = 3
K = 2
100%
75%
50%
25%
0%
K20 K17 K10 K16 K2 K1 K7 K14
M39 M50 M1 M12 M20 M47 M26 M5 M37
P47 P48 P45b P21 P18 P11 P39
F44 F30 F21 F3 F19 F40
T23 T19 T6 T39 T41 T50 T13 T26 T49
A3 A26 A31 A9 A45 A52
Kab-A
Kab-B
Pac-C
Pac-D
Tah-A
Tah-B

Fig. S8

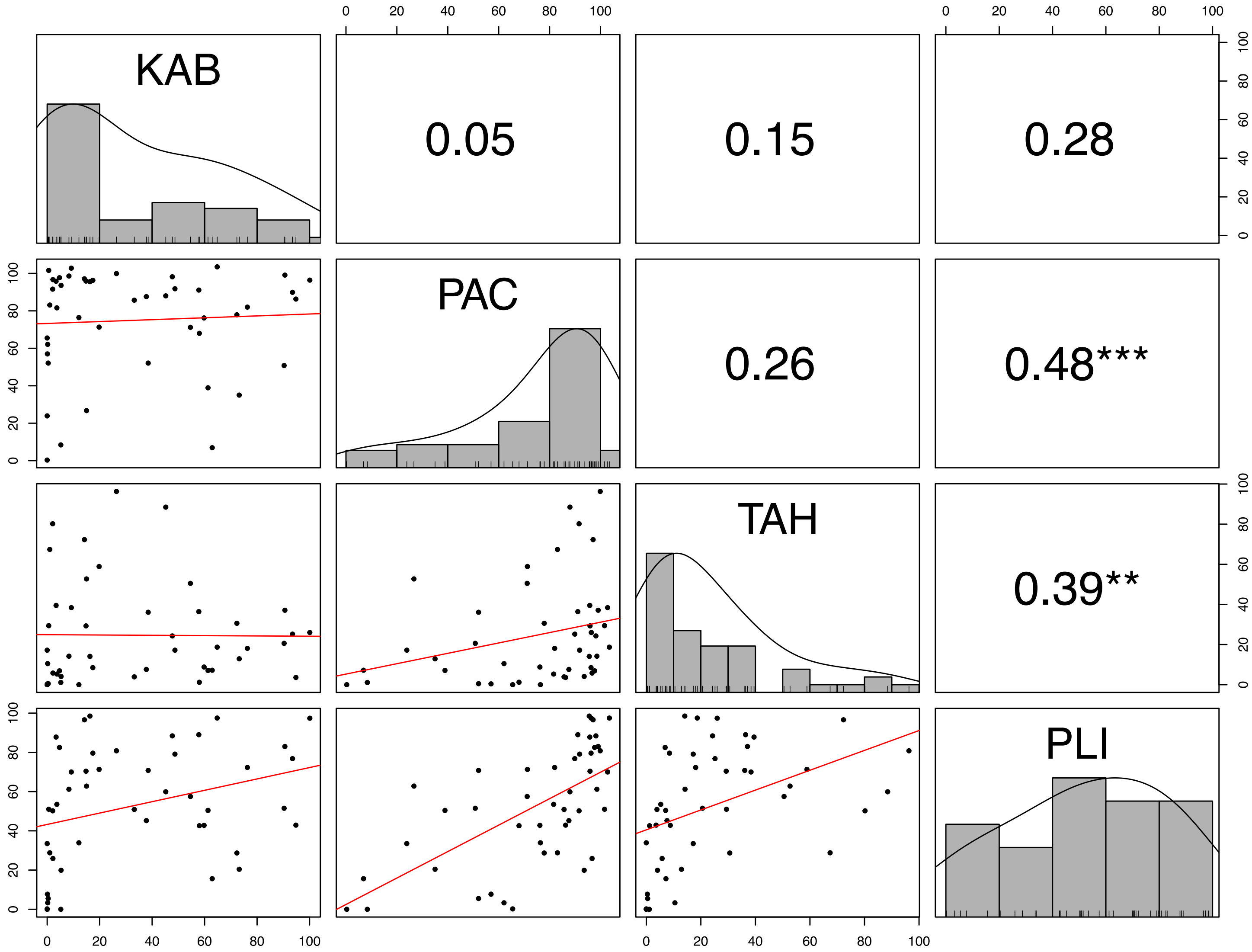

Supplement: FIGURE S1 — Power of the number of markers used to detect genotypes and measure diversity. [file Data_Sheet_1.pdf]
